# Author Correction: Autophagy-deficient macrophages exacerbate cisplatin-induced mitochondrial dysfunction and kidney injury via miR-195a-5p-SIRT3 axis

**DOI:** 10.1038/s41467-026-68421-4

**Published:** 2026-01-19

**Authors:** Yujia Yuan, Longhui Yuan, Jingchao Yang, Fei Liu, Shuyun Liu, Lan Li, Guangneng Liao, Xi Tang, Jingqiu Cheng, Jingping Liu, Younan Chen, Yanrong Lu

**Affiliations:** 1 National Health Commission (NHC) Key Laboratory of Transplant Engineering and Immunology, West China Hospital, Sichuan University, Chengdu, China; 2 Institutes for Systems Genetics, West China Hospital, Sichuan University, Chengdu, China; 3 Animal Center, West China Hospital, Sichuan University, Chengdu, China; 4 Department of Nephrology, West China Hospital, Sichuan University, Chengdu, China

**Keywords:** Mechanisms of disease, Mitochondria, Kidney, Inflammation

Correction to: *Nature Communications* 10.1038/s41467-024-47842-z, published online 23 May 2024

In the version of this article initially published, due to a figure preparation error, the H&E image labeled “NC” in Fig. 8d was inadvertently duplicated from the H&E image labeled “EXO1” in Fig. 4g. In Supplementary Fig. 5a, the image labeled “WT+Cisp” was incorrect, originating from a different experimental batch. The figure and Supplementary information are updated in the HTML and PDF versions of the article.

