# Supplementary material for: Autophagy-deficient macrophages exacerbate cisplatin-induced mitochondrial dysfunction and kidney injury via miR-195a-5p-SIRT3 axis
Source: Nat Commun. 2024 May 23;15:4383. doi: 10.1038/s41467-024-47842-z (PMC11116430; doi:10.1038/s41467-024-47842-z)
Supplement: Supplementary file 1 — Supplementary Information [file 41467_2024_47842_MOESM1_ESM.pdf]

SUPPLEMENTARY INFORMATION

Autophagy-deficient Macrophages Exacerbate Cisplatin-Induced Mitochondrial Dysfunction and Kidney Injury via miR-195a-5p-SIRT3 axis

Yujia Yuan[1†], Longhui Yuan[1†], Jingchao Yang[1], Fei Liu[1,2], Shuyun Liu[1], Lan Li[1], Guangneng Liao[4], Xi Tang[3], Jingqiu Cheng[1], Jingping Liu[1], Younan Chen[1,2]*, Yanrong Lu[1]*

[1]National Health Commission (NHC) Key Laboratory of Transplant Engineering and Immunology, West China Hospital, Sichuan University; Chengdu, 610041, China.

[2]Institutes for Systems Genetics, West China Hospital, Sichuan University; Chengdu, 610041, China.

[3]Department of Nephrology, West China Hospital, Sichuan University; Chengdu, China.

[4]Animal Center, West China Hospital, Sichuan University; Chengdu, 610041, China.

* Corresponding author. Email: chenyounan@scu.edu.cn (Y.N.C.);
luyanrong@scu.edu.cn (Y.R.L.)

† Co-first author: These authors contributed equally to this work

The supplementary file contains Supplementary Figure 1 to 9 and Supplementary Table 1 to 2.

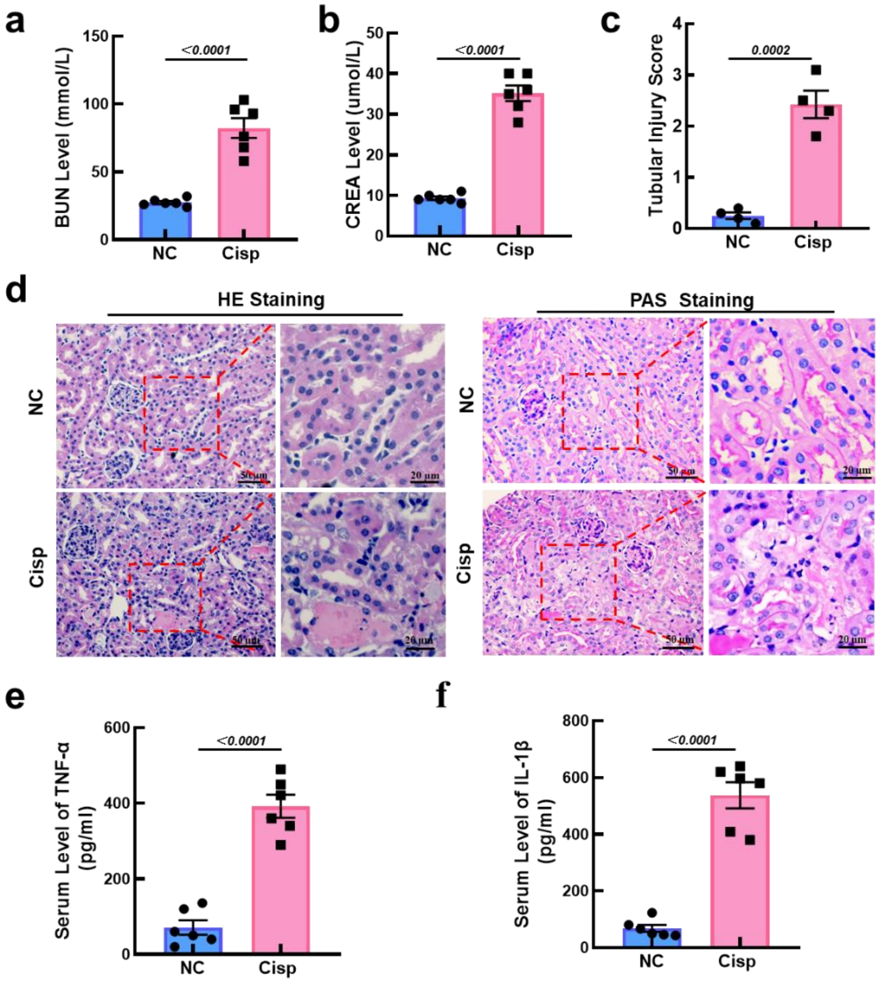

Supplementary Figure 1: Cisplatin induces kidney injury and inflammation in mice. a and **b** Serum of BUN (a) and CREA (b) in mice after injection intraperitoneally with cisplatin (16 mg/kg) for 4 days. n=6 biological replicates for each group. **c** and **d** Tubular injury score (c) and representative images (d) of hematoxylin-eosin (HE) and periodic acid-Schiff (PAS)-stained kidney sections in mice. Scale bars, 50 μm and 20 μm, n=4 biological replicates for each group. **e** and **f** The serum levels of TNF-α (e) and IL-1β (f) in cisplatin-induced AKI mice. n=6 biological replicates for each group. The data are the means ± SEMs. All statistical analysis were performed by unpaired two-tailed Student's t test. NC, normal control; Cisp, cisplatin.

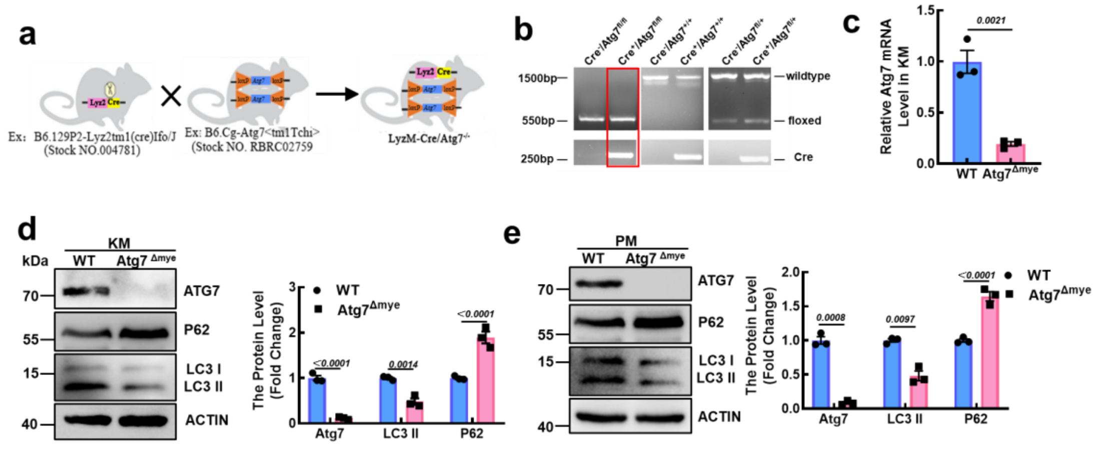

Supplementary Figure 2: Macrophages (Mφ)-specific depletion of ATG7 in Atg7$^{\Delta mye}$ mice. a Experimental scheme for generating the Atg7$^{\Delta mye}$ mice. **b** Phenotype identification of Atg7$^{\Delta mye}$ mice by PCR. **c** The ATG7 mRNA level in kidney Mφ (KM). n=3 biological replicates for each group, unpaired two-tailed Student's t test. **d** and **e** Representative images of western blot and quantitative analyses of autophagy-related genes (ATG7, P62, and LC3II) in (d) kidney Mφ (KM) and (e) peritoneal Mφ (PM) from WT and Atg7$^{\Delta mye}$ mice. ACTIN was used as the loading control. n=3 biological replicates for each group, two-way ANOVA with Tukey's multiple comparison test. The data are the means ± SEMs.

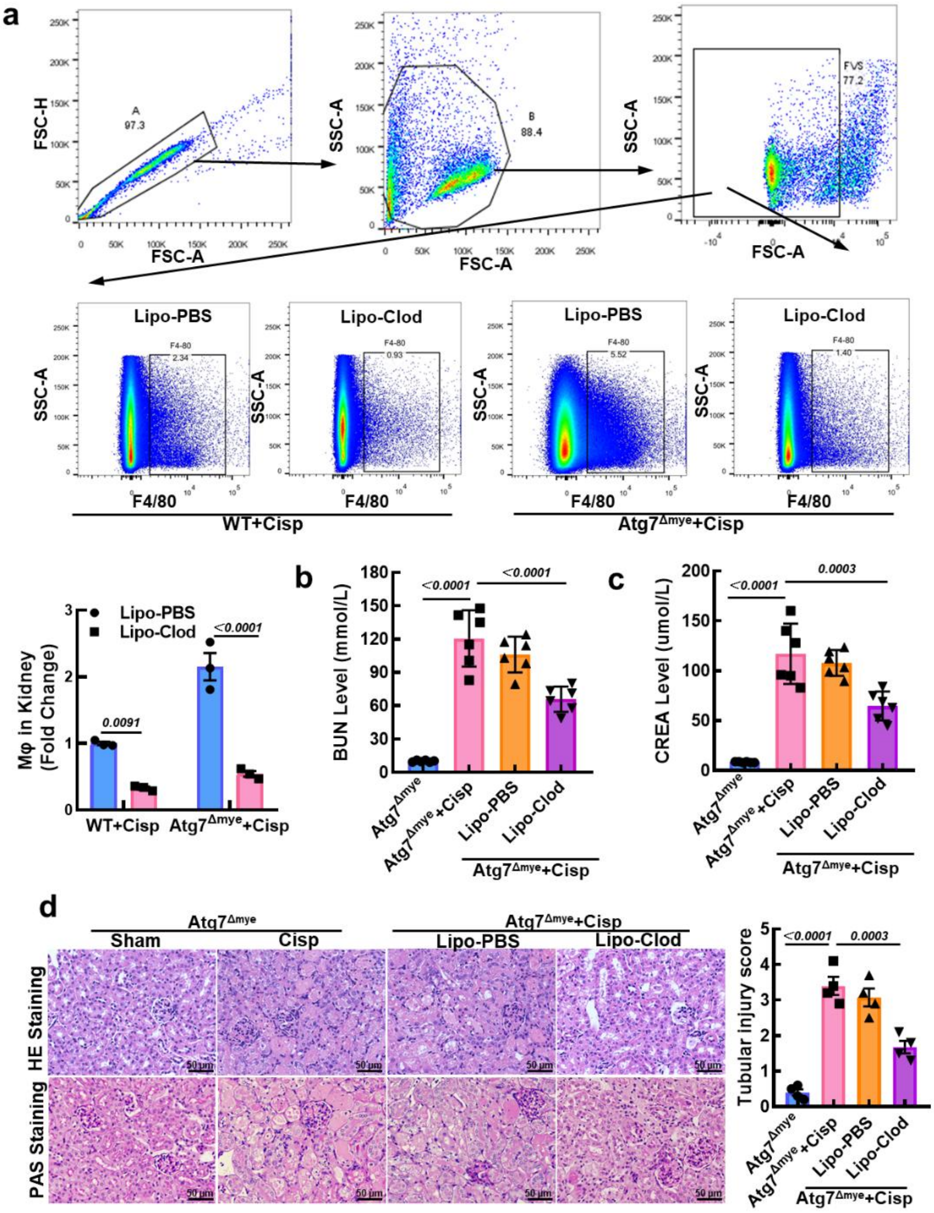

Supplementary Figure 3: Macrophages (Mφ) depletion could alleviate cisplatin-induced kidney injury in Atg7$^{\Delta mye}$ mice. a Flow cytometry analysis for Mφ depletion. n=3 biological replicates for each group, two-way ANOVA with Tukey's multiple comparison test. **b** and **c** The serum levels of BUN (b) and CREA (c) in the Atg7$^{\Delta mye}$ mice. n=6 biological replicates for each group. **d** Representative images of hematoxylin-eosin (HE) and periodic acid-Schiff (PAS)-stained kidney sections, and the tubular injury score in mice. Scale bar, 50 μm, n=4 biological replicates for each group. The data are the means ± SEMs. Statistical analysis were performed by one-way ANOVA with Tukey's multiple comparisons test in **b-d**.

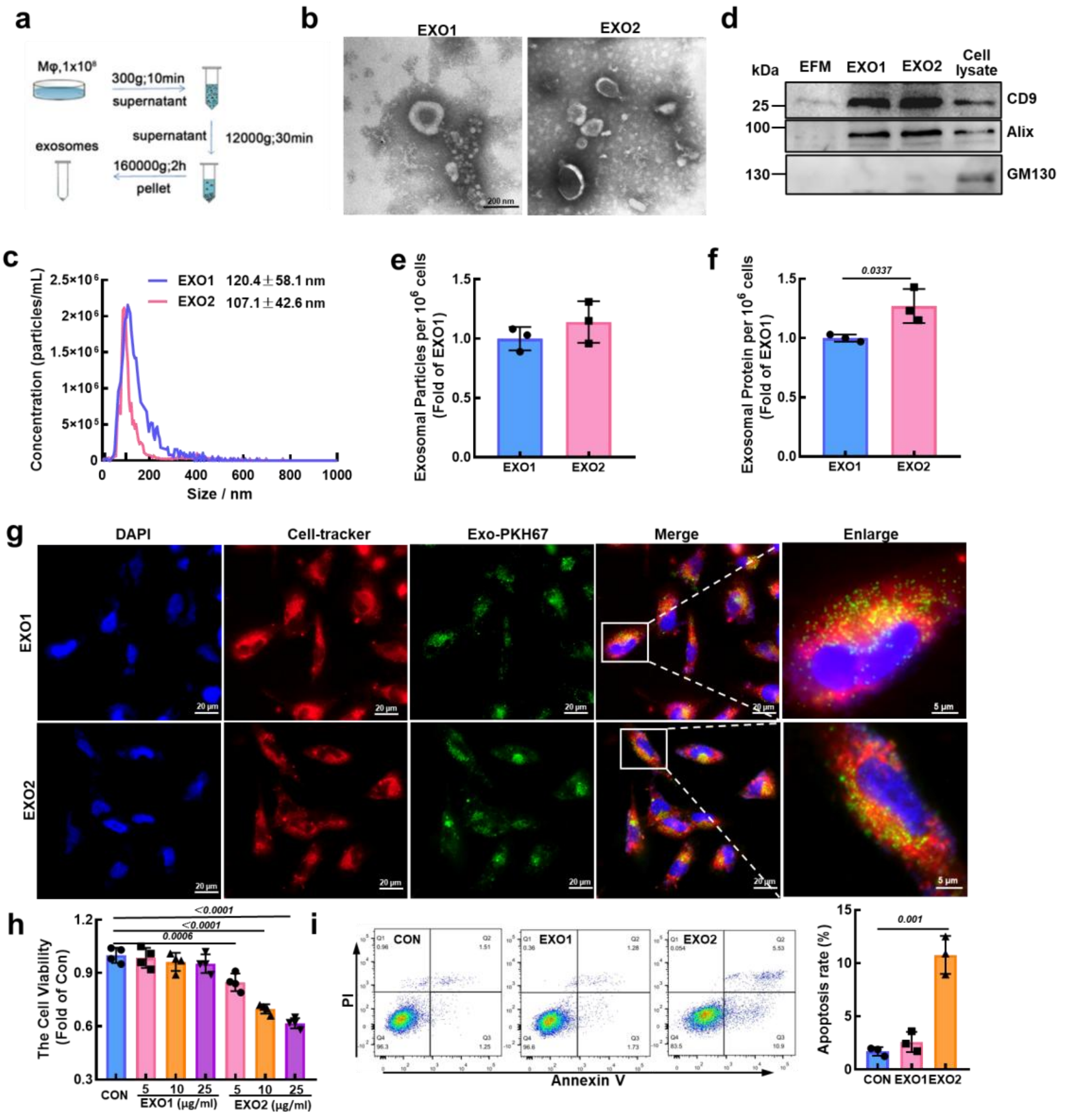

Supplementary Figure 4: Exosomes derived from ATG7 deficient-macrophages (Mφ) induces TECs injury *in vitro*. The exosomes isolated from Mφ in WT mice (Mφ^{WT}-EXO, EXO1) and Atg7$^{\Delta mye}$ mice (Mφ$^{Atg7\Delta mye}$-EXO, EXO2). **a** Schematic diagram of the methods of exosomes isolation. **b** TEM of EXO1 and EXO2. Scale bar, 200 nm. **c** NTA of EXO1and EXO2. **d** Western blot analysis of exosomal positive markers (CD9, Alix) and negative markers (GM130). **e** Exosome-sized particles and **f** total exosomal protein per million cells. n=3 biological replicates for each group, unpaired two-tailed Student's t test. **g** Fluorescence images of HK2 cells incubated with PKH67-labelled EXO2 (green). Scale bars, 20 μm and 5 μm. **h** Detection of the cell viability in HK2 cells incubated with EXO1 and EXO2 in a concentration-dependent manner. n=4 biological replicates for each group, one-way ANOVA with Dunnett's multiple comparisons test. **i** The apoptosis rate in HK2 cells after exosomes (10 μg/ml) treatment for 48 h. n=3 biological replicates for each group, unpaired two-tailed Student's t test. The data are the means ± SEMs. EXO1, Mφ^{WT}-EXO; EXO2, Mφ$^{Atg7\Delta mye}$-EXO.

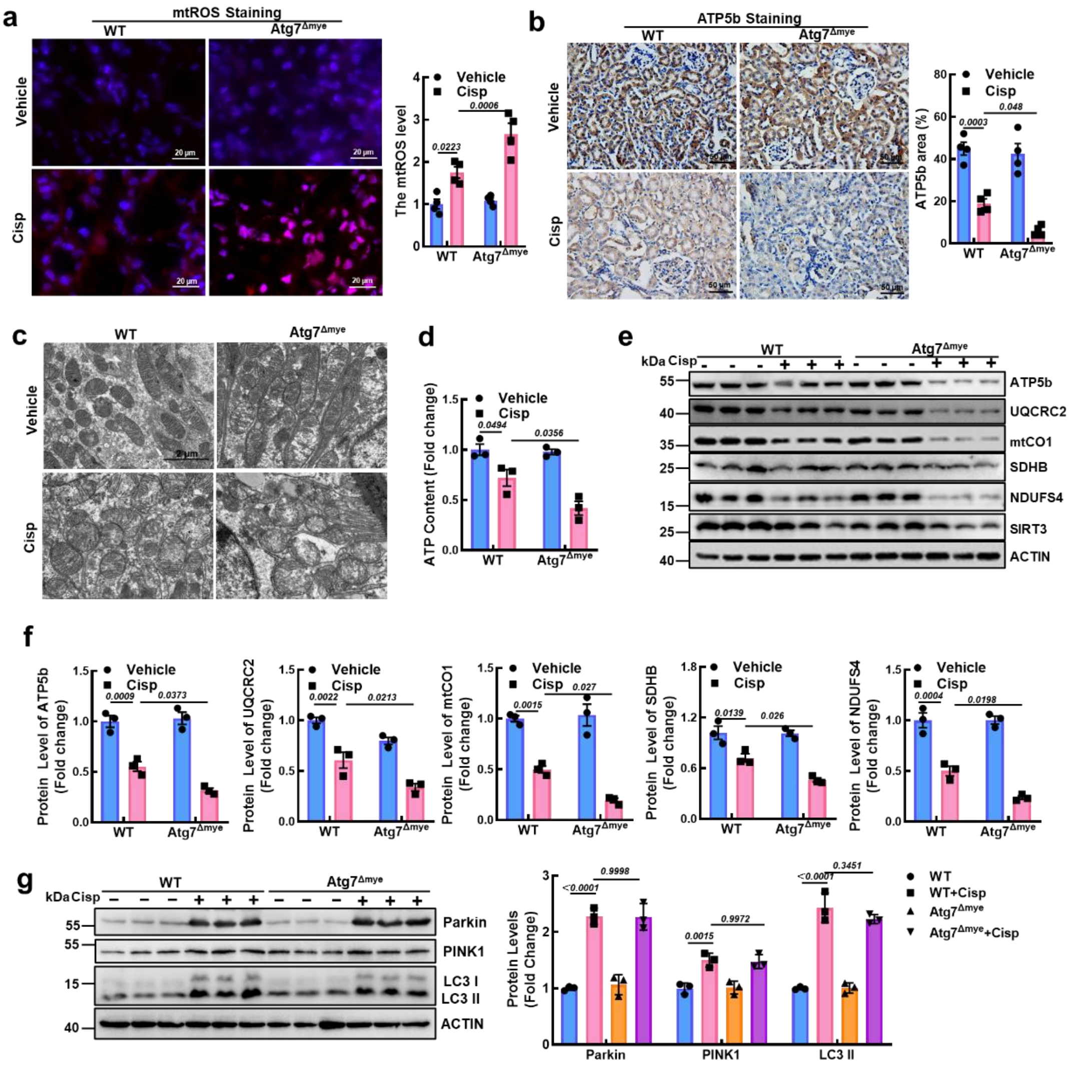

Supplementary Figure 5: Macrophages (Mφ) specific deficient of ATG7 impairs mitochondria in cisplatin-induced AKI mice. a The mitochondrial ROS (mtROS) (red) in kidney sections from WT and Atg7$^{\Delta mye}$ mice. Scale bar, 20 μm, n=4 biological replicates for each group. **b** Immunohistochemistry staining and quantification of ATP5b in paraffin- embedded sections of kidney. Scale bar, 50 μm, n=4 biological replicates for each group. **c** Representative TEM images in kidneys of mice. Scale bar, 2 μm. **d** The ATP content of kidney tissue from different groups was measured using an ATP Assay Kit, and the ATP concentration was calculated in nmol/mg protein and the data were represented as the rate of WT. n=3 biological replicates for each group. **e** and **f** Representative images of western blot (e) and quantitative analyses (f) of OXPHOS-related genes (ATP5b, UQCRC2, mtCO1, SDHB, and NDUFS4). ACTIN was used as the loading control. n=3 biological replicates for each group. **g** The expression of mitophagy-related proteins (Parkin, PINK1 and LC3 II) in cisplatin-induced Atg7$^{\Delta mye}$ mice**.** ACTIN was used as the loading control. n=3 biological replicates for each group. The data are the means ± SEMs. All statistical analysis were performed by two-way ANOVA with Tukey's multiple comparison test.

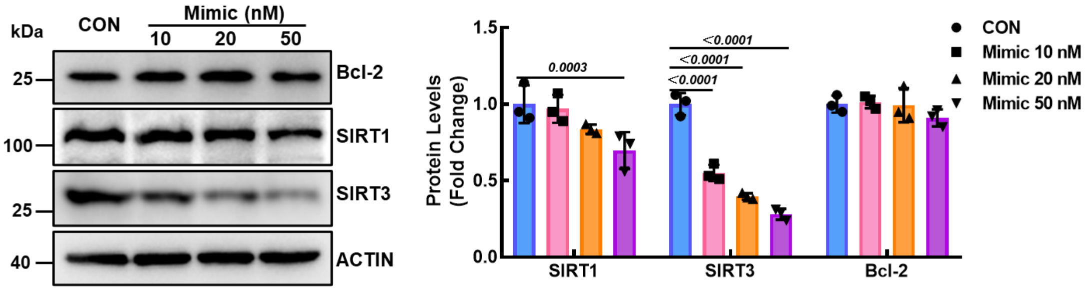

Supplementary Figure 6: The identification of miR-195a-5p target proteins. Representative images of western blot and quantitative analyses of miR-195a-5p target proteins (Bcl-2, SIRT1, and SIRT3). ACTIN was used as the loading control. n=3 biological replicates for each group, two-way ANOVA with Tukey's multiple comparison test. The data are the means ± SEMs.

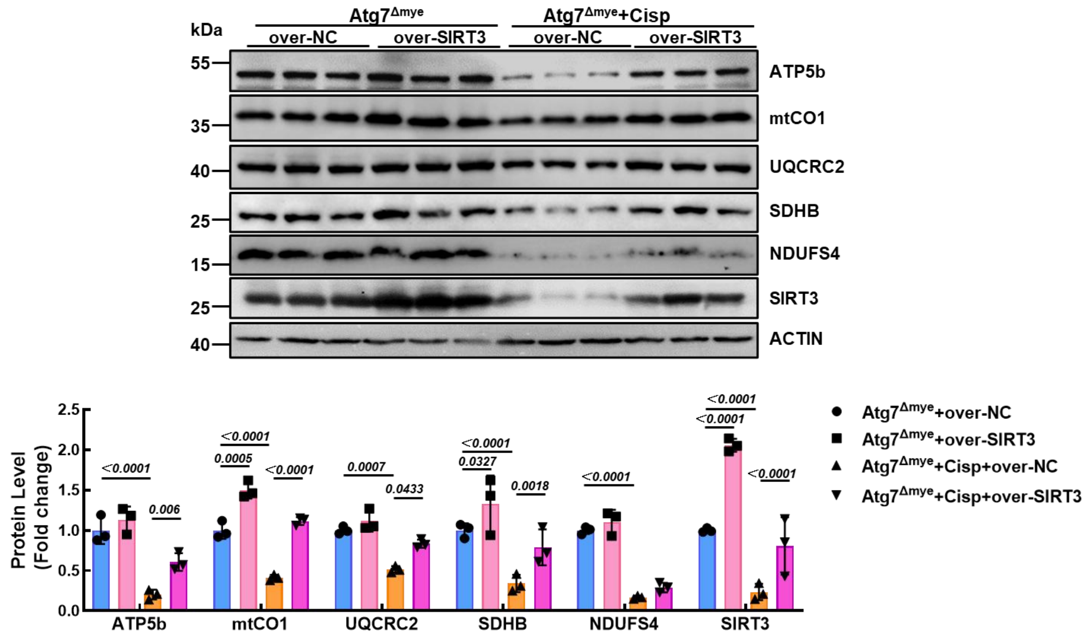

Supplementary Figure 7: Overexpression of SIRT3 alleviates cisplatin-induced the decreased of OXPHOS-related proteins. Western blot and quantitative analyses of OXPHOS-related proteins. ACTIN was used as the loading control. n=3 biological replicates for each group, two-way ANOVA with Tukey's multiple comparison test. The data are the means ± SEMs.

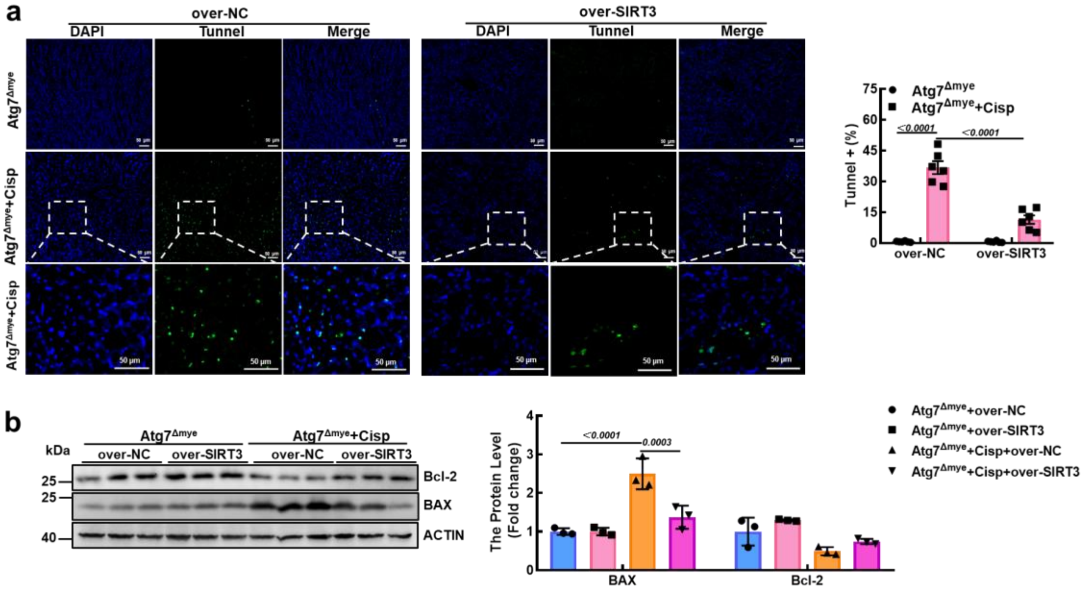

Supplementary Figure 8: Overexpression of SIRT3 alleviates cisplatin-induced kidney apoptosis in Atg7$^{\Delta mye}$ mice. a Representative micrographs and quantification of TUNEL staining (green) in each group. Scale bar, 50 µm, n=6 biological replicates for each group. **b** Representative images of western blot and quantitative analyses of BAX and Bcl-2. ACTIN was used as the loading control. n=3 biological replicates for each group. The data are the means ± SEMs. All statistical analysis were performed by two-way ANOVA with Tukey's multiple comparison test.

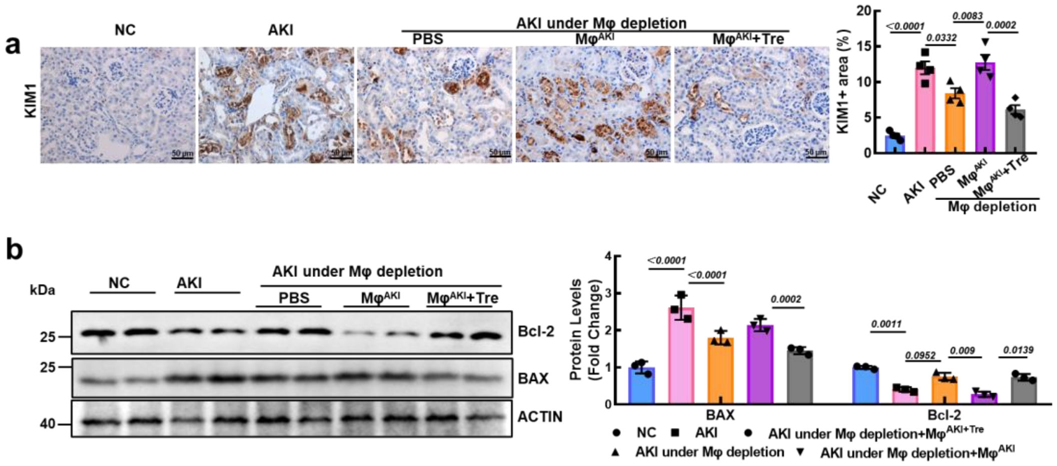

Supplementary Figure 9: Adoptive transfer of autophagy-activated macrophages (Mφ) reduces KIM1 expression and apoptosis in AKI mice. a Immunohistochemical staining and quantification of KIM1 in paraffin-embedded sections of kidney. Scale bar, 50 µm, n=4 biological replicates for each group, one-way ANOVA with Tukey's multiple comparisons test. **b** Representative images of western blot and quantitative analyses of BAX and Bcl-2. ACTIN was used as the loading control. n=3 biological replicates for each group, two-way ANOVA with Sidak's multiple comparison test. The data are the means ± SEMs.

Supplementary Table 1: Information of antibodies used in Western blot

Antibodies	Origin	Product code	Dilution
LC3B	CST	2775	1:1000
P62	Abcam	ab109012	1:10000
BECN1	CST	3495	1:1000
Atg7	CST	2631	1:500
Cleaved IL-1β	CST	63124	1:500
TNF-α	Abcam	ab255275	1:1000
BAX	Abclonal	A12009	1:1000
Bcl-2	Abcam	ab692	1:1000
CD9	Abclonal	A1703	1:500
Alix	Abcam	ab88388	1:1000
ATP5b	Abclonal	A5769	1:500
UQCRC2	Abclonal	A4181	1:500
mtCO1	Abcam	ab14705	1:1000
SDHB	Abcam	ab14714	1:1000
NDUFS4	Abclonal	A13519	1:500
SIRT3	Abcam	ab189860	1:500
PINK1	Huabio	ER1706-27	1:500
Parkin	Abclonal	A0968	1:500
ATG9	Huabio	ET1610-71	1:500
SIRT1	Abclonal	A11267	1:500
GM130	Abclonal	A11408	1:1000

Supplementary Table 2: Primers used for RT-PCR analysis

P62-F	GAACACAGCAAGCTCATCTTTC	P62-R	AAAGTGTCCATGTTTCAGCTTC
LC3-F	CCACCAAGATCCCAGTGATTAT	LC3-R	TGATTATCTTGATGAGCTCGCT
Atg7-F	GTGTACGATCCCTGTAACCTAG	Atg7-R	GATGCTATGTGTCACGTCTCTA
KIM1-F	CCTGCTGCTACTGCTCCTTGTG	KIM1-R	CCACGCTTAGAGATGCTGACTTCC
NGAL-F	CGCTACTGGATCAGAACATTTG	NGAL-R	CTTGCACATTGTAGCTCTGTAC
IL-6-F	CTCCCAACAGACCTGTCTATAC	IL-6-R	CCATTGCACAACTCTTTTCTCA
MCP-1-F	AGTTGACCCGTAAATCTGAAGC	MCP-1-R	GTGGTTGTGGAAAAGGTAGTGG
TNF-α-F	ATGTCTCAGCCTCTTCTCATTC	TNF-α-R	GCTTGTCACTCGAATTTTGAGA
IL-1β-F	CACTACAGGCTCCGAGATGAACAAC	IL-1β-R	TGTCGTTGCTTGGTTCTCCTTGTAC
CD86-F	TTGTGTGTGTTCTGGAAACGGAGTC	CD86-R	CTGCTAGGCTGATTCGGCTTCTTG
iNOS-F	CAGCACAGGAAATGTTTCAGC	iNOS-R	TAGCCAGCGTACCGGATGA
FasL-F	CCACTACCACCGCCATCACAAC	FasL-R	GCTCACGGAGTTCTGCCAGTTC
CD163-F	GCCTCTGTCATCTGCTCAGGAAAC	CD163-R	CAGCGACCACCTCCACCTACC
Arg1-F	TGCTCACACTGACATCAACACTCC	Arg1-R	TCTACGTCTCGCAAGCCAATGTAC
ACTIN-F	CTACCTCATGAAGATCCTGACC	ACTIN-R	CACAGCTTCTCTTTGATGTCAC
