# Supplementary material for: Autophagy-deficient macrophages exacerbate cisplatin-induced mitochondrial dysfunction and kidney injury via miR-195a-5p-SIRT3 axis
Source: Nat Commun. 2024 May 23;15:4383. doi: 10.1038/s41467-024-47842-z (PMC11116430; doi:10.1038/s41467-024-47842-z)
Supplement: Supplementary file 3 — Reporting Summary [file 41467_2024_47842_MOESM3_ESM.pdf]

nature portfolio

Corresponding author(s): Yanrong Lu and Younan Chen

Last updated by author(s): Mar 25, 2024

Reporting Summary

Nature Portfolio wishes to improve the reproducibility of the work that we publish. This form provides structure for consistency and transparency in reporting. For further information on Nature Portfolio policies, see our Editorial Policies and the Editorial Policy Checklist.

Statistics

For all statistical analyses, confirm that the following items are present in the figure legend, table legend, main text, or Methods section.

n/a	Confirmed	
☐	☒	The exact sample size (*n*) for each experimental group/condition, given as a discrete number and unit of measurement
☐	☒	A statement on whether measurements were taken from distinct samples or whether the same sample was measured repeatedly
☐	☒	The statistical test(s) used AND whether they are one- or two-sided<br>*Only common tests should be described solely by name; describe more complex techniques in the Methods section.*
☒	☐	A description of all covariates tested
☐	☒	A description of any assumptions or corrections, such as tests of normality and adjustment for multiple comparisons
☐	☒	A full description of the statistical parameters including central tendency (e.g. means) or other basic estimates (e.g. regression coefficient) AND variation (e.g. standard deviation) or associated estimates of uncertainty (e.g. confidence intervals)
☐	☒	For null hypothesis testing, the test statistic (e.g. *F*, *t*, *r*) with confidence intervals, effect sizes, degrees of freedom and *P* value noted<br>*Give P values as exact values whenever suitable.*
☒	☐	For Bayesian analysis, information on the choice of priors and Markov chain Monte Carlo settings
☒	☐	For hierarchical and complex designs, identification of the appropriate level for tests and full reporting of outcomes
☒	☐	Estimates of effect sizes (e.g. Cohen's *d*, Pearson's *r*), indicating how they were calculated

Our web collection on statistics for biologists contains articles on many of the points above.

Software and code

Policy information about availability of computer code

Data collection	Oxygen consumption rate (OCR) was measured using a Seahorse XF-24 flux analyzer (Seahorse Biosciences, Agilent, USA);<br>Western blot images were obtained using a ChemiDoc™ imaging system (Bio-Rad, USA);<br>Cytometry samples were analyzed using a FACSAria SORP cytometer (BD Biosciences, USA);<br>RT-PCR was measured using a Chromo4 cycler (Bio-Rad, Hercules, CA, USA);<br>Images of histology and immunohistochemistry were captured by a light microscope (Zeiss, AX10 imager A2, Oberkochen, Germany);<br>Immunofluorescence images were acquired using confocal microscopy (Nikon, TiA1-N-STORM,Tokyo, Japan);<br>The biodistribution of exosomes in the whole body and individual organs was detected by an optical imaging system (IVIS Spectrum, PerkinElmer, Waltham, MA, USA);<br>Exosome ultrastructural observation was observed via TEM (H-600, Hitachi, Ltd., Tokyo, Japan) at a voltage of 75 kV and stained kidney tissues were observed under an FEI Tecnai T20 transmission electron microscope.
Data analysis	The data were analyzed using Graphpad Prism (v9.0) and image J (v18.0). Flow cytometry analysis was performed on the CytoFLEX LX flow cytometer

For manuscripts utilizing custom algorithms or software that are central to the research but not yet described in published literature, software must be made available to editors and reviewers. We strongly encourage code deposition in a community repository (e.g. GitHub). See the Nature Portfolio guidelines for submitting code & software for further information.

Data

Policy information about availability of data

All manuscripts must include a data availability statement. This statement should provide the following information, where applicable:

- Accession codes, unique identifiers, or web links for publicly available datasets
- A description of any restrictions on data availability
- For clinical datasets or third party data, please ensure that the statement adheres to our policy

All data are available in the main text or the supplementary materials. Source data including all raw data generated in this study are provided with this paper. All data supporting the findings of this study are available from the corresponding authors upon request. Source data are provided with this paper.

Research involving human participants, their data, or biological material

Policy information about studies with human participants or human data. See also policy information about sex, gender (identity/presentation), and sexual orientation and race, ethnicity and racism.

Reporting on sex and gender	N/A
Reporting on race, ethnicity, or other socially relevant groupings	N/A
Population characteristics	N/A
Recruitment	N/A
Ethics oversight	N/A

Note that full information on the approval of the study protocol must also be provided in the manuscript.

Field-specific reporting

Please select the one below that is the best fit for your research. If you are not sure, read the appropriate sections before making your selection.

☒ Life sciences ☐ Behavioural & social sciences ☐ Ecological, evolutionary & environmental sciences

For a reference copy of the document with all sections, see nature.com/documents/nr-reporting-summary-flat.pdf

Life sciences study design

All studies must disclose on these points even when the disclosure is negative.

Sample size	The sample size was chosen based on the standards in the field. For biochemistry experiments, at least 6 mice were used. For flow cytometry analysis at least 3 mice were used. For immunohistochemistry staining at least 4 mice were used. Cell experiments were repeated at least thrice. The specific sample sizes are indicated in the main text figures, the methods sections, or the figure legends. (PMID:30206235, PMID:38461154, PMID:38467649).
Data exclusions	No data exclusions in this study.
Replication	Experiments were repeated with the same conditions and obtained similar results. A minimum of independent triplicates were carried out for each experiments as described in the legends for each figure.
Randomization	For in vivo experiments, all mice were randomly allocated into different experimental groups. For in vitro studies, randomization is not relevant as the different experimental conditions was tested using cells isolated from littermate WT and Atg7Δmye mice.
Blinding	Because the same individual is involved in the animal and cell experiments, blinding was not used for these works. But the Investigators were blinded during the sample collection and data analysis [illegible]WB, HE,PAS, IHC and IF etc.[illegible]

Reporting for specific materials, systems and methods

We require information from authors about some types of materials, experimental systems and methods used in many studies. Here, indicate whether each material, system or method listed is relevant to your study. If you are not sure if a list item applies to your research, read the appropriate section before selecting a response.

Materials & experimental systems

n/a	Involved in the study
☐	☒ Antibodies
☒	☐ Eukaryotic cell lines
☒	☐ Palaeontology and archaeology
☐	☒ Animals and other organisms
☒	☐ Clinical data
☒	☐ Dual use research of concern
☒	☐ Plants

Methods

n/a	Involved in the study
☒	☐ ChIP-seq
☐	☒ Flow cytometry
☒	☐ MRI-based neuroimaging

Antibodies

Antibodies used

Antibodies are detailed in Materials and Methods s and Supplementary Tables S1.
WB antibodies:
LC3B (Cell Signaling Technology, 2775, 1:1000), P62 (Abcam, ab109012, 1:10000), BECN1 (Cell Signaling Technology, 3495, 1:1000), Atg7 (Cell Signaling Technology, 2631, 1:500), Cleaved IL-1β (Cell Signaling Technology, 63124, 1:500), TNF-α (Abcam, ab255275, 1:1000), BAX (Abclonal, A12009, 1:1000), Bcl-2 (Abcam, ab692, 1:1000), CD9 (Abclonal, A1703, 1:500), Alix (Abcam, ab88388, 1:1000), ATP5b (Abclonal, A5769, 1:500), UQCRC2 (Abclonal, A4181, 1:500), mtCO1 (Abcam, ab14705, 1:1000), SDHB (Abcam, ab14714, 1:1000), NDUFS4 (Abclonal, A13519, 1:500), SIRT3 (Abcam, ab189860, 1:500), Parkin (Abclonal, A0968, 1:500), PINK1 (Huabio, ER1706-27, 1:500), ATG9 (Huabio, ET1610-71, 1:500), SIRT1 (Abclonal, A11267, 1:1000), GM130 (Abclonal, A11408, 1:1000).
IHC/IF antibodies:
KIM1 (RD Systems, AF1817, 1:200), TNF-α (Abcam, ab215188, 1:100), ATP5b (Abclonal, A5769, 1:200), F4/80 (Abcam, ab6640, 1:200), LC3 (Cell Signaling Technology, 2775, 1:200), Goat Anti-Rat IgG H&L (DyLight® 550) (Abcam, ab9688, 1:200).
Flow cytometry antibody:
F4/80-APC (Biolegend, 123116, BM8, 1:100), CD206-PE (Biolegend, 141706, C068C2, 1:100), CD11c-FITC (Biolegend, 117303, N418, 1:100), F4/80-PE (Biolegend, 157301, QA17A29, 1:100), LC3B (Cell Signaling Technology, E5Q2K, 1:200), Concentration-matched mouse mAb IgG2b (Cell Signaling Technology, E7Q5L), Alexa Fluor® 594 Goat anti-mouse IgG (Biolegend, 405303, Poly4053).

Validation

Antibodies are detailed in Materials and Methods and Supplementary Tables S1. Each antibody used in this study was validated by its manufacturer.
LC3B (Cell Signaling Technology, 2775, 1:1000): https://www.cellsignal.cn/product/productDetail.jsp?productId=2775
P62 (Abcam, ab109012, 1:10000): https://www.abcam.cn/products/primary-antibodies/sqstm1--p62-antibody-epr4844-autophagosome-marker-ab109012.html
BECN1 (Cell Signaling Technology, 3495, 1:1000): https://www.cellsignal.cn/product/productDetail.jsp?productId=3495
Atg7 (Cell Signaling Technology, 2631, 1:500): https://www.cellsignal.cn/product/productDetail.jsp?productId=2631
Cleaved IL-1β (Cell Signaling Technology, 63124, 1:500): https://www.cellsignal.cn/product/productDetail.jsp?productId=63124
TNF-α (Abcam, ab255275, 1:1000): https://www.abcam.cn/products/primary-antibodies/tnf-alpha-antibody-epr22598-212-ab255275.html
BAX (Abclonal, A12009, 1:1000): https://abclonal.com.cn/catalog/A12009
Bcl-2 (Abcam, ab692, 1:1000): https://www.abcam.cn/products/primary-antibodies/bcl-2-antibody-100d5-ab692.html
CD9 (Abclonal, A1703, 1:500): https://abclonal.com.cn/catalog/A1703
Alix (Abcam, ab88388, 1:1000): https://www.abcam.cn/products/primary-antibodies/alix-antibody-ab88388.html
ATP5b (Abclonal, A5769, 1:500): https://abclonal.com.cn/catalog/A5769
UQCRC2 (Abclonal, A4181, 1:500): https://abclonal.com.cn/catalog/A4181
mtCO1 (Abcam, ab14705, 1:1000): https://www.abcam.cn/products/primary-antibodies/mtco1-antibody-1d6e1a8-ab14705.html
SDHB (Abcam, ab14714, 1:1000): https://www.abcam.cn/products/primary-antibodies/sdhb-antibody-21a11ae7-ab14714.html
NDUFS4 (Abclonal, A13519, 1:500): https://abclonal.com.cn/catalog/A13519
SIRT3 (Abcam, ab189860, 1:500): https://www.abcam.cn/products/primary-antibodies/sirt3-antibody-ab189860.html
KIM1 (RD Systems, AF1817, 1:200): https://www.rndsystems.com/cn/products/mouse-tim-1-kim-1-havcr-antibody_af1817
TNF-α (Abcam, ab215188, 1:100): https://www.abcam.cn/products/primary-antibodies/tnf-alpha-antibody-epr20972-ab215188.html
F4/80 (Abcam, ab6640, 1:200): https://www.abcam.cn/products/primary-antibodies/f480-antibody-cia3-1-macrophage-marker-ab6640.html
Parkin (Abclonal, A0968, 1:500): https://abclonal.com.cn/catalog/A0968
ATG9 (Huabio, ET1610-71, 1:500): http://www.huabio.cn/product/ATG9A-antibody-ET1610-71
SIRT1 (Abclonal, A11267, 1:1000): https://abclonal.com.cn/catalog/A11267
PINK1 (Huabio, ER1706-27, 1:500): http://www.huabio.cn/product/PINK1-antibody-ER1706-27
GM130 (Abclonal, A11408, 1:1000): https://abclonal.com.cn/catalog/A11408

Goat Anti-Rat IgG H&L (DyLight® 550) (Abcam, ab9688, 1:200): https://www.abcam.cn/products/primary-antibodies/flt3-ligandflt3l-antibody-ab9688.html
F4/80-APC (Biolegend, 123116, BM8, 1:100): https://www.biolegend.com/en-us/products/apc-anti-mouse-f4-80-antibody-4071
CD206-PE (Biolegend, 141706, C068C2, 1:100): https://www.biolegend.com/en-us/products/pe-anti-mouse-cd206-mmr-antibody-7424
CD11c-FITC (Biolegend, 117303, N418, 1:100): https://www.biolegend.com/en-us/products/biotin-anti-mouse-cd11c-antibody-1814
F4/80-PE (Biolegend, 157301, QA17A29, 1:100): https://www.biolegend.com/en-us/products/purified-anti-mouse-f480-recombinant-antibody-18360
LC3B (Cell Signaling Technology, E5Q2K, 1:200): https://www.cellsignal.cn/product/productDetail.jsp?productId=83506
Concentration-matched mouse mAb IgG2b (Cell Signaling Technology, E7Q5L): https://www.cellsignal.cn/product/productDetail.jsp?productId=53484

	Alexa Fluor® 594 Goat anti-mouse IgG (Biolegend, 405303, Poly4053): https://www.biolegend.com/en-us/search-results?Clone=Poly4053

Animals and other research organisms

Policy information about studies involving animals; ARRIVE guidelines recommended for reporting animal research, and Sex and Gender in Research

Laboratory animals	C57BL/6J mice were obtained from Chengdu Dashuo Laboratory Animal Technology Co., Atg7f/f mice (Stock NO. RBRC02759; Tsukuba, Japan) with floxed alleles for the autophagy gene Atg7 were crossed with Lyz2-Cre mice (Stock NO.004781; Shanghai Nan Fang Model Organism Research Center, Shanghai, China) with the mouse lysozyme M promoter-driven Cre recombinase to generate myeloid cell specific deletion of Atg7 mice (Atg7. Mice of all genotypes were used at ages between 8 and 10 weeks. Δmye). Control mice for all experiments were littermate Atg7f/f mice lacking the Cre transgene. All mice housed in the Animal Center of West China Hospital, Sichuan University under standard conditions with free access to food and water. The light was from 7 am to 7 pm, with the temperature kept at 21-24 ? and humidity at 40-70%.
Wild animals	No wild animals were used in this study.
Reporting on sex	In general, the reason for using male mice in animal experiments is to avoid interference with the female estrous cycle. Therefore, only male mice were used in our animal studies.
Field-collected samples	No Field-collected samples were used.
Ethics oversight	Animal procedures were approved by approved by the Animal Care and Use Committee of West China Hospital, Sichuan University, and conducted according to the National Institutes of Health Guide for the Care and Use of Laboratory Animals.

Note that full information on the approval of the study protocol must also be provided in the manuscript.

Plants

Seed stocks	N/A
Novel plant genotypes	N/A
Authentication	N/A

Flow Cytometry

Plots

Confirm that:

☒ The axis labels state the marker and fluorochrome used (e.g. CD4-FITC).

☒ The axis scales are clearly visible. Include numbers along axes only for bottom left plot of group (a 'group' is an analysis of identical markers).

☒ All plots are contour plots with outliers or pseudocolor plots.

☒ A numerical value for number of cells or percentage (with statistics) is provided.

Methodology

Sample preparation	The cells were incubated with fluorescent-conjugated antibodies according to the company protocol.
Instrument	Cell fluorescence was determined using the CytoFLEX LX flow cytometer (Beckman Coulter; US).
Software	The flow cytometry results were analyzed using FlowJo™ v10.8 Software (BD Life Sciences).
Cell population abundance	We analyzed 1e4 cells in flow cytometry.
Gating strategy	Gating strategy was shown in Fig. 1e, Fig. 2b, and Fig. S3a.

☒ Tick this box to confirm that a figure exemplifying the gating strategy is provided in the Supplementary Information.
